# Association between mortality and highly antimicrobial-resistant bacteria in intensive care unit-acquired pneumonia

**DOI:** 10.1038/s41598-021-95852-4

**Published:** 2021-08-13

**Authors:** Ines Lakbar, Sophie Medam, Romain Ronflé, Nadim Cassir, Louis Delamarre, Emmanuelle Hammad, Alexandre Lopez, Alain Lepape, Anaïs Machut, Mohamed Boucekine, Laurent Zieleskiewicz, Karine Baumstarck, Anne Savey, Marc Leone, REA RAISIN Study Group, Serge Alfandari, Sébastien Bailly, Odile Bajolet, Olivier Baldesi, Anne Berger-Carbonne, Pierre-Edouard Bollaert, Cedric Bretonniere, Céline Chatelet, Philippe Corne, Isabelle Durand-Joly, Arnaud Friggeri, Gaëlle Gasan, Rémy Gauzit, Marine Giard, Caroline Landelle, Thierry Lavigne, Didier Lepelletier, Pierre-François Perrigault, Santiago Picos, Marie-Aline Robaux, Vincent Stoeckel, Jean-François Timsit, Philippe Vanhems

**Affiliations:** 1 Department of Anesthesiology and Intensive Care Unit, Aix Marseille University, Assistance Publique Hôpitaux Universitaires de Marseille, Nord Hospital, Marseille, France; 2 Department of Anesthesiology and Intensive Care Unit, University hospital of Toulouse, Toulouse, France; 3 MEPHI, IHU Méditerranée Infection, Aix Marseille Université, Marseille, France; 4 Intensive Care Unit, Centre Hospitalier Lyon Sud, Pierre Bénite, Hospices Civils de Lyon, France; 5 Rea-Raisin study group (National network for Healthcare-Associated Infection surveillance in ICU, Marseille, France; 6 APHM, EA 3279 CEReSS, School of Medicine, La Timone Medical Campus, Health Service Research and Quality of Life Center, Aix Marseille Université, Marseille, France; 7 Infection Control & Prevention, Hôpital Henry Gabrielle, Hospices Civils de Lyon, Saint Genis Laval, France; 8 PHE3ID, Centre International de Recherche en Infectiologie, INSERM U1111, CNRS Unité Mixte de Recherche 5308, ENS de Lyon, Université Claude Bernard Lyon 1, Saint Genis Laval, France; 9 Service d’anesthésie et de réanimation, Chemin des Bourrely, Hôpital Nord, 13015 Marseille, France; 10 Infection Control, Hospital of Tourcoing, Tourcoing, France; 11 University Hospital of Grenoble, Grenoble, France; 12 University Hospital of Reims, Reims, France; 13 Hospital of Aix-en-Provence, Aix-en-Provence, France; 14 Santé Publique France, Paris, France; 15 University Hospital of Nancy, Nancy, France; 16 University Hospital of Nantes, Nantes, France; 17 Hospital of Lens, Lens, France; 18 University Hospital of Montpellier, Montpellier, France; 19 Hospital of Dunkerque, Dunkerque, France; 20 Hospices Civils de Lyon, Civils de Lyon, France; 21 Assistance Publique des hôpitaux de Paris, Paris, France; 22 University Hospital of Strasbourg, Strasbourg, France; 23 Hospital of Draguignan, Draguignan, France; 24 Hospital of Bayonne, Bayonne, France; 25 Hospital of Chalons en Champagne, Chalons en Champagne, France

**Keywords:** Microbiology, Clinical microbiology, Infectious diseases, Bacterial infection

## Abstract

Data on the relationship between antimicrobial resistance and mortality remain scarce, and this relationship needs to be investigated in intensive care units (ICUs). The aim of this study was to compare the ICU mortality rates between patients with ICU-acquired pneumonia due to highly antimicrobial-resistant (HAMR) bacteria and those with ICU-acquired pneumonia due to non-HAMR bacteria. We conducted a multicenter, retrospective cohort study using the French National Surveillance Network for Healthcare Associated Infection in ICUs (“REA-Raisin”) database, gathering data from 200 ICUs from January 2007 to December 2016. We assessed all adult patients who were hospitalized for at least 48 h and presented with ICU-acquired pneumonia caused by *S. aureus, Enterobacteriaceae, P. aeruginosa,* or *A. baumannii*. The association between pneumonia caused by HAMR bacteria and ICU mortality was analyzed using the whole sample and using a 1:2 matched sample. Among the 18,497 patients with at least one documented case of ICU-acquired pneumonia caused by *S. aureus, Enterobacteriaceae, P. aeruginosa,* or *A. baumannii*, 3081 (16.4%) had HAMR bacteria. The HAMR group was associated with increased ICU mortality (40.3% *vs.* 30%, odds ratio (OR) 95%, CI 1.57 [1.45–1.70], *P* < 0.001). This association was confirmed in the matched sample (3006 HAMR and 5640 non-HAMR, OR 95%, CI 1.39 [1.27–1.52], *P* < 0.001) and after adjusting for confounding factors (OR ranged from 1.34 to 1.39, all *P* < 0.001). Our findings suggest that ICU-acquired pneumonia due to HAMR bacteria is associated with an increased ICU mortality rate, ICU length of stay, and mechanical ventilation duration.

## Introduction

Hospital-acquired pneumonia (HAP) is a common condition that is responsible for a large proportion of hospital-acquired infections, reaching 22% of cases in the United States and 15.6% of cases in France^1,2^. In intensive care units (ICUs), HAP refers to both healthcare-associated pneumonia and ventilator-associated pneumonia^3^. The attributable mortality of ventilator-associated pneumonia has been extensively evaluated in recent studies^4–6^, although this has led to conflicting results because of confounding biases^7^. Likewise, the attributable mortality of ICU-acquired pneumonia—that is, healthcare-associated pneumonia diagnosed after a 48 h stay in the ICU—remains difficult to accurately assess. However, these infections most likely have a detrimental effect on the outcomes of patients in the ICU^1,8–10^.

In ICU-acquired pneumonia, the resistance level of the causative microorganism may also affect outcome^11–13^. Investigating the relationship between antibiotic resistance and clinical outcome is challenging, as it is difficult to discriminate the confounders and determinants of this relationship. In addition, patients at the highest risk of death are also likely to be those at the highest risk of infection by highly antimicrobial-resistant (HAMR) bacteria^14^. Therefore, this study aimed to compare ICU mortality rates between patients who developed ICU-acquired pneumonia caused by HAMR bacteria and those who developed ICU-acquired pneumonia caused by non-HAMR bacteria among the following causative pathogens: *S. aureus, Enterobacteriaceae, P. aeruginosa*, and *A. baumannii.*

The first aim of our study was to compare the ICU mortality rates in patients who developed ICU-acquired pneumonia due to HAMR and non-HAMR bacteria. The secondary aims were to compare the durations of ICU stay and mechanical ventilation between these two groups.

## Material and Methods

We performed a retrospective, observational 9-year study using the REA-RAISIN database (from January 2007 to December 2016), a French national surveillance network for healthcare-associated infections in ICUs (the surveillance period was of 6 months from 2007 to 2014 and then surveillance became continuous as of 2015)^15^. The number of ICUs contributing to the database increased between 2007 and 2016, varying from 165 to 200. All the patients or their relatives were informed that their data would be used anonymously unless they disagreed with being included. This study was approved by the French Commission Nationale Informatique et Liberté (CNIL No. 588909) and Institutional Review Board (IRB No. 00009118). All included patients were followed up until death or discharge from the ICU.

The inclusion criteria were admission to an ICU for at least 48 h and diagnosis of ICU-acquired pneumonia. The diagnosis criteria for pneumonia, following the European Centre for Disease Prevention and Control (ECDC) definition, were one (if the patient had no past medical history of cardiac or pulmonary disease) or two chest radiographs showing pulmonary infiltrates and (1) at least one of the following clinical signs: hyperthermia (> 38 °C) or a leukocyte count less than 4,000 cells/mm^3^ or greater than 12,000 cells/mm^3^; (2) at least one of the following clinical criteria: onset of purulent secretions or changes in characteristics; suggestive auscultation, cough, dyspnea, or tachypnea; low oxyhemoglobin saturation; or increased pulmonary oxygen consumption; and (3) microbiological confirmation by a positive culture from directed bronchoalveolar lavage or from tracheal secretions or by an alternative method^16^. Probable cases of ICU-acquired pneumonia defined as positive according to the radiological, biological, and clinical criteria but with no positive microbiology were excluded. For each patient, we considered only the first episode of ICU-acquired pneumonia. The minimum delay between ICU admission and the onset of ICU-acquired pneumonia was 48 h. The REA-Raisin database allowed the registration of up to two causative pathogens per infection, and resistance profiles were reported only if the causative pathogens were one of the following: *S. aureus, Enterobacteriaceae, P. aeruginosa*, or *A. baumannii.*

### Data collection

Demographic and clinical data, including clinical and microbiological assessments from the electronic medical charts were analyzed. We extracted age, gender, Simplified Acute Physiology Score (SAPS) 2 at ICU admission, administration of antibiotic treatment within 48 h before or after ICU admission, length of ICU stay, patient’s provenance before ICU admission (in-hospital patient or out-hospital patient, hospitalizations occurring before the stay of interest were not recorded), immunodeficiency according to Acute Physiology and Chronic Health Evaluation II score, medical or surgical origin of patients, use of mechanical ventilation, and trauma diagnosis.

### Definition of HAMR status

Each episode of ICU-acquired pneumonia was microbiologically confirmed to identify the causal pathogens. ICU-acquired pneumonia due to HAMR bacteria was defined as the identification of at least one antimicrobial-resistant bacterium in a clinical sample, as reported in Table 1^15^.

**Table 1:**
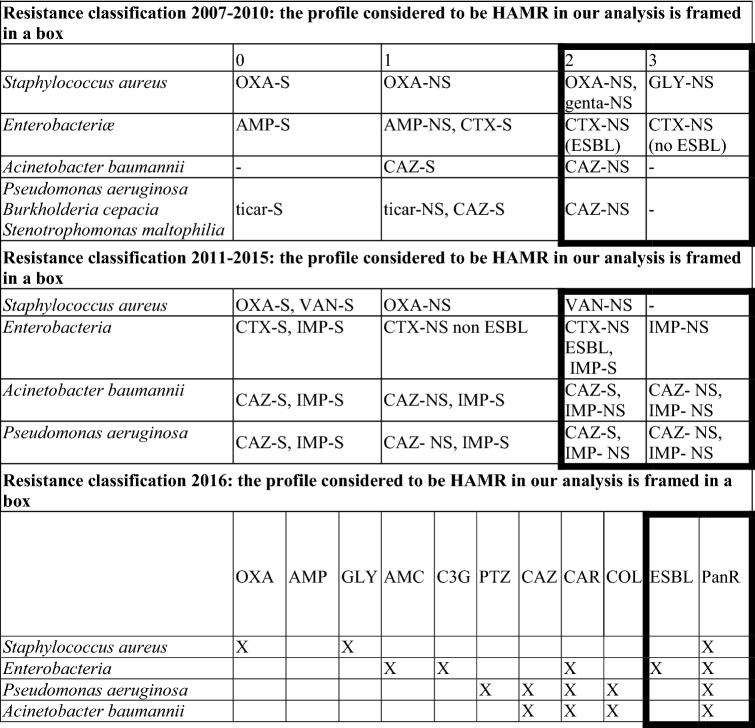
Evolution of the classification of the antimicrobial resistance by year and by micro-organisms in the REA-RAISIN database.

NS = *Non-susceptible* S = *susceptible* OXA: oxacillin (or methicillin), AMP: ampicillin (or amoxicillin), GLY: glycopeptide (vancomycin or teicoplanin), AMC: amoxicillin-clavulanic acid, ticar: ticarcillin, C3G: 3rd generation cephalosporins = cefotaxime (or ceftriaxone) PTZ: piperacillin-tazobactam, CAZ: ceftazidime, CAR: carbapenem = imipenem or doripenem or meropenem, IMP: imipenem, VAN: vancomycin, COL: colistin, ESBL: extended-spectrum beta-lactamase-producing, PANR:non susceptible to all tested agents, HAMR: highly antimicrobial-resistant.

### Statistical methods

To assess the association between HAMR bacteria and ICU mortality, analyses were conducted in two steps: (1) on the whole sample and (2) on a 1:2 matched sample (at least one control). Matching was based on seven factors: sex, age (within 5 years), SAPS 2 (within 10 points), antibiotic treatment at admission (yes–no), category of patient (medical *vs.* surgical), mechanical ventilation and type of pathogen. The last factor was determined as follows: when pneumonia was caused by a single pathogen (n = 15,717) or two identical pathogens (n = 991), patients were matched based on the same causative pathogen; for pneumonia caused by two different pathogens (n = 2096), a Delphi review was performed by the authors and a panel of experts to determine on which pathogens the matching should be based. The results of the Delphi review are provided in the Supplementary Material. Patients were matched using the %match SAS macro^17^, which implements an optimal matching algorithm^18^. The optimal algorithm sorts cases and controls, identifies all pairs that satisfy the specified distance measures, and then selects the set of pairs that minimizes the total distance between all pairs.

For each sample, patients with pneumonia due to HAMR bacteria were compared to patients with pneumonia due to non-HAMR according to the main characteristics. To assess the link between HAMR status and ICU mortality, comparisons based on socio-demographic, clinical, and hospital data between survivors and non-survivors were performed (1) on the whole sample using chi2 tests or Student’s t tests according to the nature of the variable and (2) on the matched sample using conditional logistic regression^19^, taking into account the matched procedure. Odds ratios (OR) with a 95% confidence interval (CI) were estimated. Multivariate models were assessed to confirm the effect of HAMR status on ICU mortality after adjusting for the main confounding factors (1) with the whole sample using logistic regression (adjustment for sex, age, SAPS 2, antibiotic at admission, immunodeficiency status, mechanical ventilation, traumatic situation, provenance, category of patients, delay of pneumonia) and (2) with the matched sample using a generalized linear model, PROC GLIMMIX SAS (adjustment for provenance, immunodeficiency, trauma, delay of pneumonia). The link between HAMR status and ICU mortality was also assessed in predefined subgroups: men and women, younger (< 65 years) and older (≥ 65 years) patients, medical and surgical patients, mechanical ventilation and no mechanical ventilation, and patients with or without antibiotics at ICU admission. A two-sided p-value of less than 0.05 was considered to indicate statistical significance. All statistical analyses were conducted using IBM SPSS Statistics for Windows (Version 21.0. Armonk, NY: IBM Corp) and SAS 9.4 (SAS Institute).

## Results

**Figure 1:**
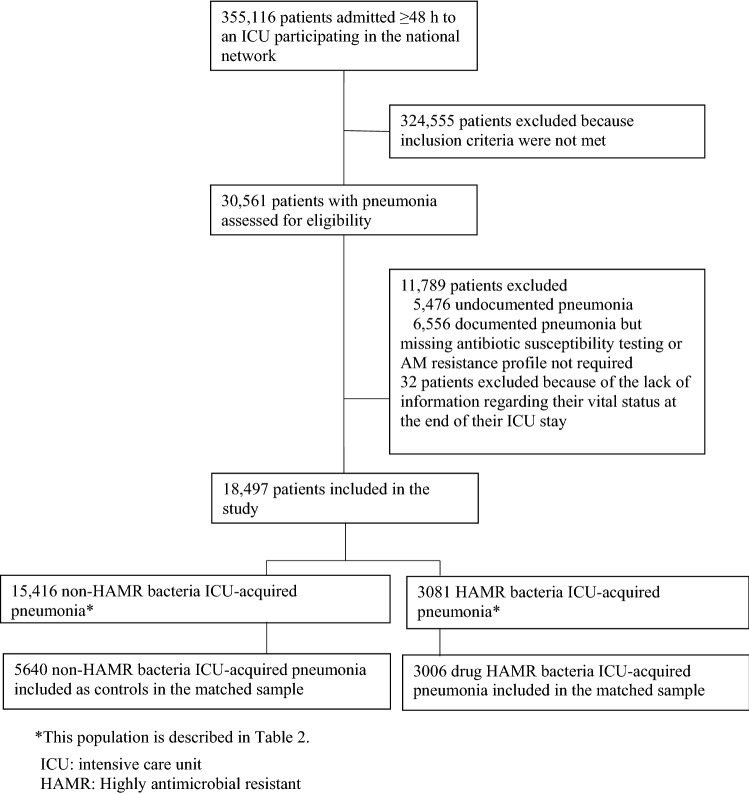
Selection of the final study group (n = 18,772) from 355,116 patients hospitalized ≥ 48 h in French ICUs, 2007–2016.

For the study period of 9 years, the database contained 355,116 patients, of which 30,561 (8.6%) developed at least one episode of ICU-acquired pneumonia. A total of 25,096 patients had a documented infection, and for 18,529, a bacteria profile of the isolated strains corresponding to *S. aureus*, *Enterobacteriaceae*, *P. aeruginosa*, or *A. baumannii* was available. Of these 18,529 patients, the vital status of 18,497 was available at the time of discharge from the ICU. A flowchart of the study is displayed in Fig. 1, and the patient features are presented in Table 2.

**Table 2:** Patient characteristics and comparison according to the HAMR status.

	Patients with vital status at discharge					
	Whole sample		*P*-value	Matched sample		*P*-value
	Non HAMR	HAMR		Non HAMR	HAMR	
	n = 15,416	n = 3081		n = 5640	n = 3,006	
**Age (years)**						
M ± SD	62.2 ± 16.4	64.0 ± 14.9	< 0.001	64.7 ± 13.6	64.2 ± 14.7	_
m [IQR]	65 [53–75]	66 [56–75]		67 [58–75]	66 [56–75]	
**Sex**						
Female	4318 (28.0)	842 (27.3)	0.442	1506 (26.7)	807 (26.8)	_
Male	11,098 (72.0)	2239 (72.7)		4134 (73.3)	2199 (73.2)	
**Provenance**						
Inpatient	6897 (44.9)	1677 (54.5)	< 0.001	2735 (48.6)	1642 (54.7)	< 0.001
Outpatient	8474 (55.1)	1398 (45.5)		2897(51.4)	1361 (45.3)	
**Type**						
Medical	10,170 (66.1)	2216 (72.2)	< 0.001	4083 (72.4)	2174 (72.3)	_
Surgery	5216 (33.9)	854 (27.8)		1556 (27.6)	831 (27.7)	
**SAPS 2 at admission**						
M ± SD	50.5 ± 18.2	52.4 ± 18.2	< 0.001	51.3 ± 15.4	52.2 ± 17.6	_
m [IQR]	49 [37–62]	50 [39–64]		50 [40–60]	50 [39–64]	
**Immunosuppression**						
No	12,816 (86.2)	2357 (78.4)	< 0.001	4673 (85.0)	2310 (78.5)	< 0.001
Yes	2058 (13.8)	650 (21.6)		821 (14.9)	632 (21.5)	
**Mechanical ventilation**						
No	319 (2.1)	58 (1.9)	0.505	62 (1.1)	38 (1.3)	_
Yes	15,089 (97.9)	3020 (98.1)		5578 (98.9)	2968 (98.7)	
**Mechanical ventilation (days)**						
M ± SD	27.3 ± 24.5	31.5 ± 25.9	< 0.001	29.2 ± 25.1	31.5 ± 25.8	< 0.001
m [IQR]	21 [12–35]	24 [15–41]		22 [13–37]	24 [15–41]	
**Trauma patients**						
No	12,967 (84.3)	2881(93.6)	< 0.001	4990 (88.5)	2818 (93.7)	< 0.001
Yes	2416 (15.7)	197 (6.4)		646 (11.5)	188 (6.3)	
**Antibiotic treatment at admission**						
No	6137 (40.2)	683 (22.3)	< 0.001	1283 (22.8)	668 (22.3)	_
Yes	9131 (59.8)	2381 (77.7)		4345 (77.2)	2331 (77.7)	
**ICU duration (days)**						
M ± SD	32.9 ± 26.2	37.2 ± 26.7	< 0.001	35.1 ± 27.6	37.3 ± 26.6	< 0.001
m [IQR]	26 [16–41]	30 [19–48]		28 [18–43]	30 [19–48]	
**Delay between ICU admission and event**						
M ± SD	12.8 ± 12.3	15.9 ± 12.3	< 0.001	14.1 ± 14.4	16.0 ± 12.4	< 0.001
m [IQR]	10 [6–16]	13 [8–19]		11 [7–17]	13 [8–19]	
**Delay between pneumonia and ICU discharge (days)**						
M ± SD	21.1 ± 21.7	22.3 ± 22.1	< 0.001	22.0 ± 22.3	22.3 ± 22.1	0.391
m [IQR]	15 [8–26]	16 [8–29]		15 [9–27]	16 [8–29]	
**ICU mortality**						
Survivors	10,785 (70.0)	1840 (59.7)	< 0.001	3799 (67.4)	1794 (59.7)	< 0.001
Non-survivors	4631 (30.0)	1241 (40.3)		1841 (32.6)	1212 (40.3)	

*ICU* intensive care unit, *HAMR* high antimicrobial resistance, *SAPS2* simplified acute physiology score. M ± SD: mean ± standard deviation; m [IQR]: median [interquartile range].

*with vital status not missing.

Of the 18,497 included cases of infection, 3081 (17%) were infected with HAMR bacteria and 15,416 (83%) with non-HAMR bacteria (details about the pathogens are provided in the Supplemental Material). The ICU mortality rate was 32%, representing 5872 patients aged 68 ± 13 years with an average SAPS 2 of 55 ± 18. The average ICU length of stay was 33 ± 26 days. Invasive mechanical ventilation was required in 18,109 (98%) patients for a duration of 28 ± 25 days. Of note, 11,512 (62%) patients received antibiotics within 48 h of admission. The reasons for ICU admission were medical (67%) and surgical (33%).

For several sociodemographic and clinical variables, there were significant differences between patients with ICU-acquired pneumonia due to HAMR bacteria and those with ICU-acquired pneumonia due to non-HAMR bacteria (Table 2). Therefore, 5640 non-HAMR ICU-acquired pneumonia cases were matched with 3006 ICU-acquired cases of pneumonia caused by HAMR bacteria. Details are provided in Table 2.

**Figure 2:**
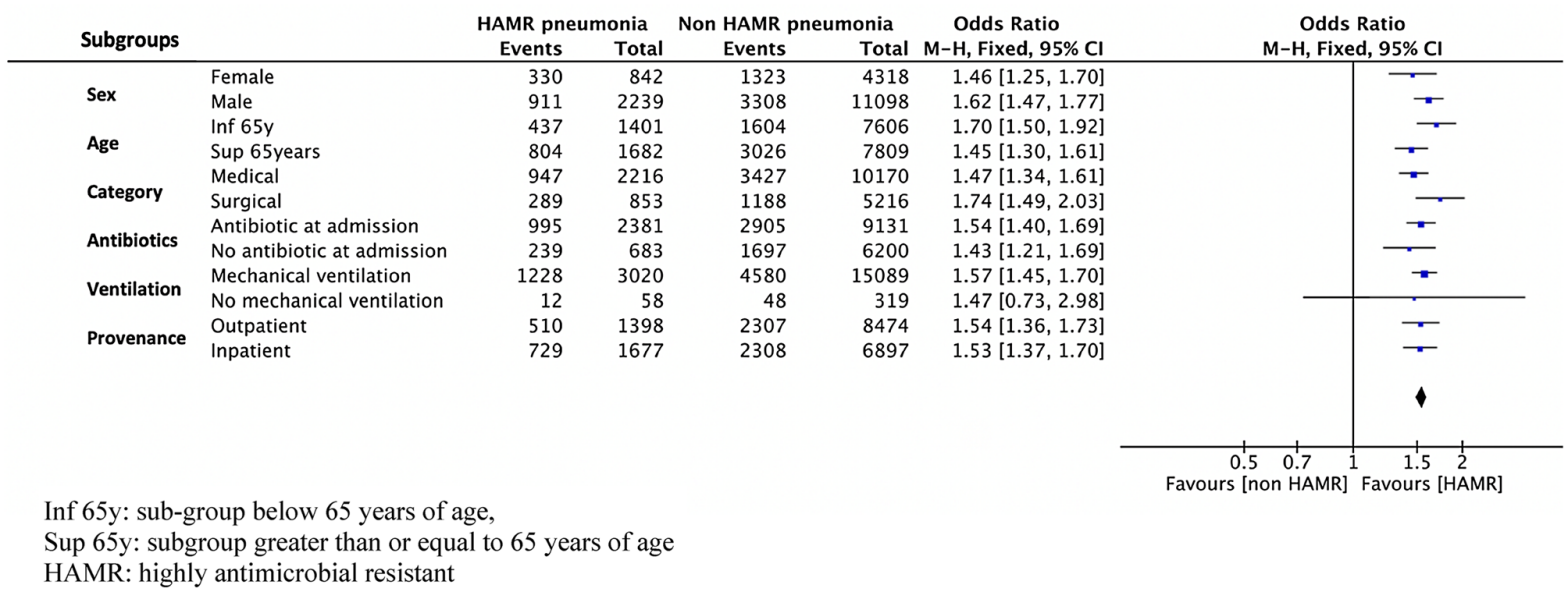
Risk of mortality associated with HAMR status by subgroup.

In the whole sample, HAMR group and non-HAMR group were associated with 40.3% and 30.0% ICU mortality rates, respectively (differential 10, odds ratio (OR) and 95% confidence interval (CI) 1.57[1.45–1.70], *P* < 0.001). Age, sex, provenance, immunosuppression, ICU length of stay, and HAMR status were associated with ICU mortality (Table 3). HAMR status was still associated with ICU mortality (1) in the matched sample (OR 95%, CI 1.39 [1.27–1.52], *P* < 0.001) (Table 3); (2) after adjusting for the main confounding factors (ORs ranged from 1.34 to 1.39, all p-values < 0.001) (Table 4); and (3) in prespecified subgroups: females *versus* males, age below 65 years *versus* above (or equal) 65 years, antibiotic at ICU admission *versus* no-antibiotic at ICU admission, medical patient *versus* surgical patient, mechanical ventilation *versus* no mechanical ventilation, and in-hospital patient *versus* out-hospital patient (Fig. 2).

**Table 3:** Factors associated with ICU mortality on the whole sample and the matched sample (univariate analysis).

	Whole sample*			
	Survivors	Non-survivors	OR [95%CI]**	*P*-value
	n = 12,625	n = 5872		
**Age (years)**				
M ± SD	60.0 ± 16.8	67.8 ± 13.4	1.03 [1.03–1.04]	< 0.001
m [IQR]	62 [51–73]	70 [60–78]		
**Sex**				
Female	3507 (27.8)	1653 (28.2)	0.98 [0.91–1.05]	0.599
Male (1)	9118 (72.2)	4219 (71.8)		
**Provenance**				
Inpatient	5537 (44.0)	3037 (51.9)	0.72 [0.68–0.77]	< 0.001
Outpatient (1)	7055 (56.0)	2817 (48.1)		
**Type**				
Medical	8012 (63.6)	4374 (74.8)	0.58 [0.55–0.63]	< 0.001
Surgery (1)	4592 (36.4)	1477 (25.2)		
**SAPS 2 at admission**				
M ± SD	48.7 ± 18.0	55.2 ± 18.1	1.02 [1.01–1.02]	
m [IQR]	47 [36–60]	54 [42–67]		
**Immunosuppression**				
No	10,655 (87.3)	4518 (79.6)	1.79 [1.62–1.92]	< 0.001
Yes (1)	1547 (12.7)	1161 (20.4)		
**ICU duration (days)**				
M ± SD	34.5 ± 25.9	31.7 ± 27.3	1.00 [0.99–1.00]	< 0.001
m [IQR]	28 [18–43]	25 [15–40]		
**Delay between ICU admission and event**				
M ± SD	12.8 ± 10.9	14.4 ± 14.8	1.01 [1.00–1.01]	< 0.001
m [IQR]	10 [6–16]	11 [7–18]		
**Delay between event and ICU discharge**				
M ± SD	22.7 ± 21.8	18.3 ± 21.5	0.99 [0.98–0.99]	< 0.001
m [IQR]	16 [9–29]	12 [6–23]		
**Mechanical ventilation**				
No	317 (2.5)	60 (1.0)	2.49 [1.88–3.29]	< 0.001
Yes (1)	12,301 (97.5)	5808 (99.0)		
**Mechanical ventilation duration (days)**				
M ± SD	27.9 ± 24.9	28.3 ± 24.8	1.00 [1.00–1.00]	0.344
m [IQR]	21 [12–35]	22 [13–36]		
**Trauma patients**				
No	10,436 (82.8)	5412 (92.4)	0.39 [0.35–0.43]	< 0.001
Yes (1)	2169 (17.2)	444 (7.6)		
**Antibiotic treatment at ICU admission**				
No	4912 (39.2)	1908 (32.9)	1.31 [1.23–1.40]	< 0.001
Yes (1)	7612 (60.8)	3900 (67.1)		
**Bacteria feature**				
Non HAMR	10,785 (85.4)	4631 (78.9)	1.57 [1.45–1.70]	< 0.001
HAMR (1)	1840 (14.6)	1241 (21.1)		

	Matched sample*			
	Survivors	Non-survivors	OR [95%CI]**	*P*-value
	n = 5593	n = 3053		
**Bacteria feature**				
Non HAMR	3799 (67.4)	1794 (59.7)	1.39 [1.27–1.52]	< 0.001
HAMR (1)	1841 (32.6)	1212 (40.3)		

*ICU* intensive care unit, *HAMR* highly antimicrobial resistant *SAPS2* simplified acute physiology score.

M ± SD: mean ± SD; m [IQR]: median [interquartile range]; OR [95%CI]: odd ratio [95% confidence interval].

*with vital status not missing; **OR is provided for the modality (1).

**Table 4:** Highly antimicrobial resistant status and ICU mortality (variate analysis).

Whole sample				Matched sample			
Model adjusted for…		OR [95%CI]	*P*-value	Model adjusted for…		OR [95%CI]	*P*-value
1	Sex, age, SAPS2	1.48 [1.36–1.61]	< 0.001	1	Provenance	1.39 [1.27–1.53]	< 0.001
2	Sex, age, SAPS2, antibiotic at admission,	1.45 [1.34–1.58]	< 0.001	2	Immunosuppression	1.37 [1.24–1.51]	< 0.001
3	Sex, age, SAPS2, antibiotic at admission, immunodeficiency, mechanical ventilation	1.41 [1.29–1.53]	< 0.001	3	Provenance, immunosuppression	1.36 [1.23–1.50]	< 0.001
4	Sex, age, SAPS2, antibiotic at admission, immunodeficiency, mechanical ventilation, traumatic situation, provenance, category of patient	1.36 [1.25–1.48]	< 0.001	4	Provenance, immunosuppression, trauma	1.34 [1.21–1.48]	< 0.001
5	Sex, age, SAPS2, antibiotic at admission, immunodeficiency, mechanical ventilation,traumatic situation, provenance, delay of pneumonia	1.35 [1.24–1.47]	< 0.001	5	Provenance, immunosuppression, trauma, delay of pneumonia	1.33 [1.20–1.46]	< 0.001

*SAPS2* simplified acute physiology score.

The mean durations of ICU length of stay (37 ± 26 days *versus* 33 ± 26 days, *P* < 0.001) and mechanical ventilation (31 ± 26 days *versus* 27 ± 24 days, *P* < 0.001) were higher in the HAMR group than in the non-HAMR group. The delay between ICU admission and the pneumonia onset differed between HAMR group and non-HAMR group from 16.0 days ± 12.4 to 14.1 days ± 14.4, respectively (*P* < 0.001).

## Discussion

According to our results, developing ICU-acquired pneumonia due to HAMR bacteria was an independent risk factor for ICU mortality. To our knowledge, our study included one of the largest cohorts to assess the association between infection due to HAMR bacteria and ICU mortality.

Lambert et al. published the largest prospective European study (n = 119,699 patients, of whom 8525 were diagnosed with HAP)^20^. They concluded that the effect of antimicrobial resistance on mortality was modest. In the same line, Paramythiotou et al. concluded that a direct association between infections caused by Gram-negative resistant bacteria and ICU mortality was not confirmed^21^. However, most studies were performed in single centers and included small numbers of patients. In addition, most were characterized by a high degree of heterogeneity that prevented definitive conclusions from being made^21^. Three studies have suggested that antibiotic resistance led to an increase in crude mortality, even after adjusting for two of them^12,14,22^. Here, we found an association between ICU mortality and the occurrence of an infection due to HAMR bacteria.

The effect of bacterial resistance on patient outcomes can be explained by three determinants. First, patients infected by HAMR bacteria are more likely to receive an inadequate empirical antimicrobial therapy^23^. As there is an association between the adequateness of the empirical antimicrobial therapy and survival, this hypothesis may explain the increased ICU mortality that we reported here^24^. Second, an increased virulence has been suspected in some resistant bacteria^25^, as suggested by a murine model of infection due to *P. aeruginosa*^26^. The authors found that the acquisition of antibiotic resistance improved the fitness of the bacteria and thus promoted its survival and virulence. However, the higher virulence of HAMR bacteria remains unlikely as conversely, other studies described a loss of potency and virulence in specific bacteria-antibiotic pairs^27,28^. The third determinant relates to host factors and co-morbidities. A frail patient has a higher risk of recurrent hospitalizations, exposure to antibiotics, and thus colonization and infection by HAMR bacteria^29^. Moreover, the effects of antibiotics themselves could be deleterious, as suggested previously^30^.

Our study has several limitations. First, this was a retrospective analysis of a large database. Hence, our choices for the statistical approach could be a matter of debate. However, our results were confirmed using several statistical approaches. Second, the definition of ICU-acquired pneumonia relies on each on-site physician, with different sampling techniques, without external confirmation, while the diagnosis of HAP remains challenging^31^. Third, only *S. aureus*, *Enterobacteriaceae*, *P. aeruginosa*, and *A. baumannii* pneumonia were included in the analysis, thus excluding, notably, streptococci and other gram-negative bacteria. Finally, there was no mention in the database of the antimicrobial therapy, specifically the adequacy and delay of the empirical treatment. As discussed above, this is a major determinant of mortality in these patients^23,24^. However, in our study, the ICU-acquired pneumonia due to HAMR bacteria occurred later than those due to non-HAMR bacteria, suggesting an increased number of late-onset pneumonia in the HAMR group. Following international guidelines, the patients with late-onset pneumonia are more prone to receive broad-spectrum antibiotics than those with early-onset pneumonia^31^. Notably, 62.2% of the patients included in our study received antibiotics at admission in the ICU. This finding is in line with the rates recently reported in an international observational 24-h point prevalence among 15,202 patients^32^. The selection of our population was based on voluntary participation in the network and a duration of ICU stay of at least 48 h (for a reduced surveillance workload). Thus, our findings may not be reflective of the entire ICU patient population, as they may pertain specifically to patients exposed to infections acquired in the ICU. Finally, the definition of antimicrobial-resistant bacteria evolved during the study period, which could have affected our findings, despite efforts to ensure comparability across the nine years. As ecology and therapies have evolved over the years with the arrival of new molecules in our therapeutic arsenal, the classification of resistances, established prospectively by the designers of the database, has also evolved towards a more recent and precise definition in 2016 which is more consistent with recent guidelines^1,31^.

## Conclusion

In conclusion, the findings of our study suggest that ICU-acquired pneumonia due to HAMR bacteria was associated with an increased ICU mortality rate, duration of ICU stay, and mechanical ventilation duration. However, the reasons behind this association remain to be elucidated.

## Supplementary Information

Supplementary Information.

## Data Availability

This study was approved by the French Commission Nationale Informatique et Liberté (CNIL No. 588909) and Institutional Review Board (IRB No. 00009118). Our study has no attached data.

## References

[1] Kalil AC (2016). Management of adults with hospital-acquired and ventilator-associated pneumonia: 2016 clinical practice guidelines by the infectious diseases society of America and the American thoracic society. Clin. Infect. Dis..

[2] Daniau C (2017). Infections associées aux soins en établissement de santé: résultats de l’enquête nationale de prévalence 2017, france/healthcare-associated infections in healthcare facilities: results of french national point prevalence survey. Science.

[3] Timsit J-F, Esaied W, Neuville M, Bouadma L, Mourvillier B (2017). Update on ventilator-associated pneumonia. F1000Res.

[4] Timsit J-F, Zahar J-R, Chevret S (2011). Attributable mortality of ventilator-associated pneumonia. Curr. Opin. Crit. Care.

[5] Bekaert M (2011). Attributable mortality of ventilator-associated pneumonia: a reappraisal using causal analysis. Am. J. Respir. Crit. Care Med..

[6] Nguile-Makao M (2010). Attributable mortality of ventilator-associated pneumonia: respective impact of main characteristics at ICU admission and VAP onset using conditional logistic regression and multi-state models. Intensive Care Med..

[7] Papazian L, Klompas M, Luyt C-E (2020). Ventilator-associated pneumonia in adults: a narrative review. Intensive Care Med..

[8] Torres A (2017). International ERS/ESICM/ESCMID/ALAT guidelines for the management of hospital-acquired pneumonia and ventilator-associated pneumonia. Eur. Respir. J..

[9] Ibn SW (2019). A comparison of the mortality risk associated with ventilator-acquired bacterial pneumonia and nonventilator ICU-acquired bacterial pneumonia. Crit. Care Med..

[10] Melsen WG (2013). Attributable mortality of ventilator-associated pneumonia: a meta-analysis of individual patient data from randomised prevention studies. Lancet. Infect. Dis.

[11] Damas P (2011). Severity of ICU-acquired pneumonia according to infectious microorganisms. Intensive Care Med..

[12] Razazi K (2017). Frequency, associated factors and outcome of multi-drug-resistant intensive care unit-acquired pneumonia among patients colonized with extended-spectrum β-lactamase-producing Enterobacteriaceae. Ann. Intensive Care.

[13] Zahar J-R (2005). Is Methicillin resistance associated with a worse prognosis in *Staphylococcus* aureus ventilator-associated pneumonia?. Clin. Infect. Dis..

[14] Bottazzi A (2018). Mortality attributable to different Klebsiella susceptibility patterns and to the coverage of empirical antibiotic therapy: a cohort study on patients admitted to the ICU with infection. Intensive Care Med..

[15] Lepape A, MacHut A, Savey A (2018). National network REA-RAISIN of adult intensive care acquired infections methods and main results. Med. Intensive Reanim..

[16] Plachouras D, Lepape A, Suetens C (2018). ECDC definitions and methods for the surveillance of healthcare-associated infections in intensive care units. Intensive Care Med..

[17] Bergstralh EJ, Kosanke JL (1995). Computerized matching of cases to controls. Tech. Rep..

[18] Rosenbaum PR (1989). Optimal matching for observational studies. J. Am. Stat. Assoc..

[19] Hosmer DW, Lemeshow S, Sturdivant RX (2013). Applied Logistic Regression.

[20] Lambert ML (2011). Clinical outcomes of health-care-associated infections and antimicrobial resistance in patients admitted to European intensive-care units: a cohort study. Lancet. Infect. Dis.

[21] Paramythiotou E, Routsi C (2016). Association between infections caused by multidrug-resistant gram-negative bacteria and mortality in critically ill patients. World J. Crit. Care Med..

[22] Barbier F (2016). Colonization and infection with extended-spectrum β-lactamase-producing *Enterobacteriaceae* in ICU patients: What impact on outcomes and carbapenem exposure?. J. Antimicrob. Chemother..

[23] Rottier WC, Ammerlaan HSM, Bonten MJM (2012). Effects of confounders and intermediates on the association of bacteraemia caused by extended-spectrum β-lactamase-producing enterobacteriaceae and patient outcome: a meta-analysis. J. Antimicrob. Chemother..

[24] Rhodes A (2017). Surviving Sepsis campaign: international guidelines for management of sepsis and septic shock: 2016. Intensive Care Med..

[25] Guillard T, Pons S, Roux D, Pier GB, Skurnik D (2016). Antibiotic resistance and virulence: understanding the link and its consequences for prophylaxis and therapy. BioEssays News Rev. Mol. Cell. Dev. Biol..

[26] Roux D (2015). Fitness cost of antibiotic susceptibility during bacterial infection. Sci. Transl. Med..

[27] Hraiech S (2013). Impaired virulence and fitness of a colistin-resistant clinical isolate of *Acinetobacter* baumannii in a rat model of pneumonia. Antimicrob. Agents Chemother..

[28] Andersson DI, Hughes D (2010). Antibiotic resistance and its cost: is it possible to reverse resistance?. Nat. Rev. Microbiol..

[29] Giarratano A, Green SE, Nicolau DP (2018). Review of antimicrobial use and considerations in the elderly population. Clin. Interv. Aging.

[30] Jensen JU (2011). Procalcitonin-guided interventions against infections to increase early appropriate antibiotics and improve survival in the intensive care unit: a randomized trial. Crit. Care Med..

[31] Torres A (2017). International ERS/ESICM/ESCMID/ALAT guidelines for the management of hospital-acquired pneumonia and ventilator-associated pneumonia. Eur. Respir. J..

[32] Vincent J-L (2020). Prevalence and outcomes of infection among patients in intensive care units in 2017. JAMA.

