# Supplementary material for: Association between mortality and highly antimicrobial-resistant bacteria in intensive care unit-acquired pneumonia
Source: Sci Rep. 2021 Aug 13;11:16497. doi: 10.1038/s41598-021-95852-4 (PMC8363636; doi:10.1038/s41598-021-95852-4)
Supplement: Supplementary file 1 — Supplementary Information. [file 41598_2021_95852_MOESM1_ESM.docx]

**eTable 1. Bacteria isolated in ICU-acquired pneumonia and their resistance profiles**

|  | **Overall** |
| --- | --- |
| **Total number of exploitable episodes** | 18,529 |
| Polymicrobial episodes (2 germs) | 2,922 |
| Of which germs from different classes (see Delphi review) | 1,524 |
| **Total number of isolated bacteria with documented resistance profile** | 21,389 |
| **Gram-positive bacteria** |  |
| *Staphylococcus aureus* | 4,817 (22.5%) |
| Non highly resistant *S. aureus* | 4,734 (22.1%) |
| Highly resistant *S. aureus* | 83 (0.4%) |
|  |  |
| **Gram-negative bacteria** | 10,673 (49.9%) |
| *Enterobacter spp* | 2,442 (11.4%) |
| Non highly resistant *Enterobacter spp* | 1,776 (8.3%) |
| Highly-resistant *Enterobacter spp* | 666 (3.1%) |
|  |  |
| *Escherichia coli* | 2,944 (13.8%) |
| Non highly resistant *E. coli* | 2,542 (11.9%) |
| Highly resistant *E. coli* | 402 (1.9%) |
|  |  |
| Klebsiella spp | 2,232 (10.4%) |
| Non highly resistant Klebsiella spp | 1,678 (7.8%) |
| Highly resistant Klebsiella spp | 554 (2.6%) |
|  |  |
| Other Enterobacteriaceae | 3,055 (14.3%) |
| Non highly resistant | 2,857 (13.4%) |
| Highly resistant | 198 (0.9%) |
|  |  |
| **Non-fermenting, gram-negative bacteria** | 5,899 (27.6%) |
| *Pseudomonas aeruginosa* | 5,331 (24.9%) |
| Non highly resistant *P. aeruginosa* | 4,334 (20.3%) |
| Highly resistant *P. aeruginosa* | 997 (4.7%) |
|  |  |
| *Acinetobacter baumannii* | 568 (2.7%) |
| Non highly resistant *A. baumannii* | 289 (1.4%) |
| Highly resistant *A. baumannii* | 279 (1.3%) |

**eTable 2. Delphi review results**

|  | **When pneumonia is due to two germs, one being S.aureus, and the other belonging to the group of enterobacteria** | | |
| --- | --- | --- | --- |
| **Matching should be performed on** | *S.aureus* | Enterobacteria | Patient exclusion |
| Number of votes | 2 | 5 | 2 |
|  | **When pneumonia is due to two germs, one being P.aeruginosa, and the other belonging to the group of enterobacteria** | | |
| **Matching should be performed on** | *P.aeruginosa* | Enterobacteria | Patient exclusion |
| Number of votes | 8 | 1 | 1 |
|  | **When pneumonia is due to two germs, one being P.aeruginosa, and the other S.aureus** | | |
| **Matching should be performed on** | *P.aeruginosa* | *S.aureus* | Patient exclusion |
| Number of votes | 8 | 0 | 2 |
|  | **When pneumonia is due to two germs, one being A.baumanii and the other belonging to the group of enterobacteria** | | |
| **Matching should be performed on** | *A.baumanii* | Enterobacteria | Patient exclusion |
| Number of votes | 7 | 0 | 3 |
|  | **When pneumonia is due to two germs, one being A.baumanii and the other S.aureus** | | |
| **Matching should be performed on** | *A.baumanii* | *S.aureus* | Patient exclusion |
| Number of votes | 6 | 1 | 3 |

A Delphi review was conducted among the authors and a group of experts to determine to determine on which pathogens the matching should be performed on. Ten voters were invited to answer the questions presented above in bold. The solution chosen was the one that received the highest number of votes.
